# HDAC and Proteasome Inhibitors Synergize to Activate Pro-Apoptotic Factors in Synovial Sarcoma

**DOI:** 10.1371/journal.pone.0169407

**Published:** 2017-01-05

**Authors:** Aimée N. Laporte, Jared J. Barrott, Ren Jie Yao, Neal M. Poulin, Bertha A. Brodin, Kevin B. Jones, T. Michael Underhill, Torsten O. Nielsen

**Affiliations:** 1 Faculty of Medicine, Vancouver Coastal Health Research Institute, University of British Columbia, Vancouver, British Columbia, Canada; 2 Department of Orthopaedics, Huntsman Cancer Institute, University of Utah, Salt Lake City, Utah, United States of America; 3 Department of Oncology and Pathology, Karolinska Institutet, Stockholm, Sweden; 4 Department of Cellular and Physiological Sciences, Biomedical Research Centre, University of British Columbia, Vancouver, British Columbia, Canada; The Ohio State University, UNITED STATES

## Abstract

Conventional cytotoxic therapies for synovial sarcoma provide limited benefit, and no drugs specifically targeting its driving SS18-SSX fusion oncoprotein are currently available. Patients remain at high risk for early and late metastasis. A high-throughput drug screen consisting of over 900 tool compounds and epigenetic modifiers, representing over 100 drug classes, was undertaken in a panel of synovial sarcoma cell lines to uncover novel sensitizing agents and targetable pathways. Top scoring drug categories were found to be HDAC inhibitors and proteasomal targeting agents. We find that the HDAC inhibitor quisinostat disrupts the SS18-SSX driving protein complex, thereby reestablishing expression of *EGR1* and *CDKN2A* tumor suppressors. In combination with proteasome inhibition, HDAC inhibitors synergize to decrease cell viability and elicit apoptosis. Quisinostat inhibits aggresome formation in response to proteasome inhibition, and combination treatment leads to elevated endoplasmic reticulum stress, activation of pro-apoptotic effector proteins BIM and BIK, phosphorylation of BCL-2, increased levels of reactive oxygen species, and suppression of tumor growth in a murine model of synovial sarcoma. This study identifies and provides mechanistic support for a particular susceptibility of synovial sarcoma to the combination of quisinostat and proteasome inhibition.

## Introduction

Synovial sarcoma is an aggressive, high-grade soft tissue tumor arising most frequently in the extremities of adolescents and young adults [1]. Conventional cytotoxic therapy, including doxorubicin and ifosphamide, provides limited benefit. Following surgery and radiation, patients remain at high risk for both early and late metastases, and despite best available therapies the mortality rate remains approximately 50% within 10 years of diagnosis [2].

Synovial sarcoma is characterized by a fusion oncogene derived from the chromosomal translocation t(X;18)(p11.2;q11.2) [3]. This translocation results in the fusion of the N-terminus of *SS18* to the C-terminus of *SSX1*, *SSX2* or *SSX4*, resulting in the chimaeric oncoprotein SS18-SSX. SS18 has been described as a member of the SWI/SNF complex involved in nucleosome remodeling, while SSX proteins (normally expressed only in adult testes and at low levels in the thyroid) can act as transcriptional corepressors [4–6].

Although neither SS18 nor SSX possesses DNA binding domains, interactions with transcriptional and epigenetic regulators allow SS18-SSX to elicit significant deregulation of gene expression [7]. The fusion protein has been proposed to interact in place of native SS18, leading to aberrant SWI/SNF-mediated gene transcription [8]. We have shown that SS18-SSX acts as a bridge connecting activating transcription factor 2 (ATF2) to transducin-like enhancer of split 1 (TLE1), proteins which otherwise do not associate [9]. ATF2 binds DNA at CRE/ATF sites, where under normal conditions it functions as a histone acetyltransferase (HAT) to increase gene transcription in response to cellular stress signals [10]. TLE1 has been observed to interact with polycomb-repressor complex 2 (PRC2) and histone deacetylases (HDAC), and has been shown to mediate formation of a complex in which PRC2/HDAC1 represses gene expression at SS18-SSX targeted loci [9, 11]. When this association is disrupted by specific knockdown of TLE1, ATF2 or SS18-SSX, synovial sarcoma cell lines undergo apoptosis, indicating this complex association is important for tumor cell survival [9].

Observed targets for SS18-SSX-mediated repression include early-growth factor 1 (*EGR1*), a key positive regulator of tumor suppressor PTEN, and *CDKN2A*, important for normal cell cycle regulation [9, 12, 13]. In addition, upregulation of the PI3K/AKT/mTOR growth signaling pathway as well as increased expression of the pro-apoptotic protein BCL-2 (B-cell lymphoma-2) are hallmarks of synovial sarcoma biology, together eliciting a proliferative and apoptosis-resistant phenotype [13, 14].

HDAC inhibitors have been shown to disrupt the SS18-SSX/TLE1/ATF2 protein complex [9, 11], providing an explanation for the sensitivity of synovial sarcoma cells to HDAC inhibition [15]. Though no drugs able to specifically target the SS18-SSX fusion oncoprotein are currently available, compounds able to disrupt its partnering interactions might be expected to reactivate normal gene transcription and elicit anti-tumor effects and clinical response. In this study, we undertook a high-throughput drug screen of over 900 compounds representing 100 drug classes, in an effort to identify sensitizing agents and targetable pathways in synovial sarcoma that might expand treatment options. From this, the potency of HDAC inhibition in synovial sarcoma was reinforced, with specific effects on the oncoprotein and consequent gene reactivation demonstrated in a panel SS18-SSX-containing cell lines. In addition, an effective combination treatment with proteasome inhibitors was uncovered. The combination of HDAC and proteasome inhibition synergizes to activate pro-apoptotic factors and bring about cell death and suppresses tumor growth in a murine model of synovial sarcoma, presenting a strong candidate strategy for clinical benefit in synovial sarcoma.

## Materials and Methods

### Cell culture and chemicals

The following human synovial sarcoma cell lines were kindly provided: SYO-1 [16] (Dr. Akira Kawai, National Cancer Centre Hospital, Tokyo, Japan), FUJI [17] (Dr. Kazuo Nagashima, Hokkaido University School of Medicine, Sapporo, Japan), YaFuss [18], and HS-SY-II [19] (Dr. Scott Lowe, Memorial Sloan Kettering Cancer Centre, New York, USA), SSR3A1 (murine), and MoJo [20] (Dr. K. Jones, University of Utah, Salt Lake City, UT), Yamato-SS [21], and ASKA-SS [21] (Dr. K. Itoh, Osaka Medical Center for Cancer and Cardiovascular Diseases, Japan). Sarcoma cell lines were maintained in RPMI-1640 medium supplemented with 10% fetal bovine serum (FBS) (Life Technologies, Waltham, MA, USA) and the presence of a disease-defining *SS18-SSX* fusion oncogene was confirmed by RT-PCR analysis. As additional human non-sarcoma controls, breast cancer cell line MCF7 (ATCC HTB22) and human embryonic kidney HEK293T (ATCC CRL3216) were purchased from the ATCC (Manassas, VA, USA) and cultured in DMEM medium with 10% FBS. Patient-derived primary synovial sarcoma (83-SS) and matched muscle cells (83-muscle) were obtained from a surgical specimen in accordance with ethics guidelines and approval from Regionala Etikprovningsnämden, Stockholm (No. 2013/1979-31/3). The cells were dissociated by enzymatic digestion using 0.01% collagenase (Sigma-Aldrich, St. Louis, MI, USA). The dispersed cells were grown in DMEM/F12 media containing 10% FBS. Muscle cells were grown in muscle-specific growth media (PromoCell, Heidelberg, Germany). The outgrowing synovial sarcoma primary cells were confirmed for *SS18-SSX* expression by RT-PCR analysis. All cells were grown at 37°C, 95% humidity, and 5% CO_2_. Pharmacologic compounds were purchased from Selleck Chemicals (Houston, TX, USA).

### High-throughput drug screen assay

A 900 compound library composed of over 100 different classes of tool compounds and epigenetic modifiers from the Ontario Institute of Cancer Research (OICR, Toronto, ON, Canada) together with epigenetic modifiers (Cayman Biochemical, Ann Arbor, MI, USA, Item 11076) (S1 Fig) were screened on six synovial sarcoma cell lines and two unrelated control cell lines (MCF7 and HEK293T). Cells were seeded in 96-well plates at 1e4 cells/well. The following day, compounds from the drug library were transferred from stock plates (1 mM in DMSO) using a 96-pin tool with a diameter of 0.4-mm, to bring about a final concentration of ~1 μM per well. Plates were developed with MTS reagent 48 hours post treatment and viability assessed relative to vehicle-only controls (0.1% DMSO). For each synovial sarcoma cell line, compounds bringing about a decrease in relative viability of greater than 90% were scored as 1 (+++), 75.1–90% as 0.5 (++), 50–75% as 0.25 (+), and less than 50% as 0 (-). The total score across the six cell lines was calculated as a sum to a maximum score of 6. Control cell lines MCF7 and HEK293T were screened concurrently to demonstrate potential drug specificity against synovial sarcoma. A viability heatmap was created using the Gene-E software program (Broad Institute, Cambridge, MA, USA). Top hits were validated in a dose-response curve and IC_50_ values were calculated.

### Western blots

Protein was collected following 24 hour treatments with indicated compounds. Samples were separated by 10% SDS-PAGE and transferred to PVDF membranes (Bio-Rad Laboratories, Hercules, CA, USA). Blots were incubated with indicated antibodies; Santa-Cruz Biotechnology (Dallas, TX, USA): SS18 sc-28698 1:200, GAPDH sc-25778 1:1500, BIK (NBK) sc-305625 1:500, BIM sc-374358 1:500, BCL-2 sc-492 1:250, p-BCL-2 sc-101762 1:250, HDAC6 sc-11420 1:250, vinculin sc-5573 1:5000, α-tubulin sc-8035 1:200; Cell Signaling (Danvers, MA, USA): EGR1 4153S 1:1000, Ac-α-tubulin 5335 1:1000, HDAC1 5356 1:1000, LC3B 2775 1:1000, p-PERK 3179S 1:1000, ER stress antibody kit 9956 (PERK, IRE1α, BiP, CHOP) 1:1000; Abcam (Cambridge, MA, USA): p16INK4a ab108349 1:500, p14ARF ab124282 1:500. Signals were visualized using the Odyssey Infrared System (LI-COR Biosciences, Lincoln, NE, USA).

### Proximity Ligation Assay (PLA)

Cells were seeded in culture-treated chamber slides at 3e4 cells/well. The following day, wells were treated with indicated compounds for 12 hours. Cells were then washed twice with PBS, fixed with 4% formaldehyde and permeabilized with 0.1% Triton X-100. Wells were blocked with blocking buffer and incubated overnight at 4°C with primary antibodies at a 1:1000 dilution: SS18 (Santa-Cruz Biotechnology, Dallas, TX, USA, sc-28698), TLE1 (Origene Technologies, Rockville, MD, USA, TA800301). Proximity ligation was performed utilizing the Duolink® In Situ Red Starter Kit Mouse/Rabbit (Sigma-Aldrich, Oakville, ON, Canada) according to the manufacturer’s protocol. The oligonucleotides and antibody-nucleic acid conjugates used were those provided in the Sigma-Aldrich PLA kit (DUO92101). Alexa Fluor 488 secondary antibody (Life Technologies, Waltham, MA, USA) was included during the final incubation of the PLA protocol. Fluorescence was detected using a Zeiss Axioplan 2 microscope at 40x. PLA images were quantified in triplicate using ImageJ software (NIH, Bethesda, MD, USA) as foci per nucleus, defined as the number of interaction points counted per nucleus.

### Real-time qPCR

Total RNA was isolated from treated cells using the RNeasy Mini kit (Qiagen, Valencia, CA, USA) and was then reverse transcribed to cDNA using Oligo(dT) (Invitrogen, Waltham, MA, USA) and Superscript III (Invitrogen, Waltham, MA, USA). SYBR Green (Roche, Mississauga, ON, Canada) reagent was used for qPCR expression analysis, using an ABI ViiA7 qPCR system. The following primers for expression were used; *EGR1*: sense: AGCCCTACGAGCACCTG, antisense: GGTGGGTTGGTCATG; *CDKN2A*: sense: CAACGCACCGAATAGTTACGG, antisense: AACTTCGTCCTCCAGAGTCGC. All transcript levels were normalized to *GAPDH* RNA expression as well as to DMSO treated conditions to calculate relative fold induction of expression using the comparative Ct (ΔΔCt) method.

### Cell viability assays

Cells were seeded in 96-well plates at 1e4 cells/well and treated in triplicate at indicated doses of the tested compounds. IC_50_ doses were determined in synovial sarcoma cell lines by dose curve studies. Cell viability was assessed in the cell lines as compared with the vehicle condition (0.1% DMSO) at 48 hours post treatment using MTS reagent (Promega, Madison, WI, USA). The primary cells were seeded in 384-well plates at 1e3 cells/well, and viability was assessed by quantifying ATP levels using the CellTitre-Glo luminescence-based assay (Promega, Madison, WI, USA), as compared with the vehicle condition (0.1% DMSO) at 48 hours post treatment. Cell confluency and apoptosis induction was assessed over a 48-hour timeframe utilizing the IncuCyte Zoom® live cell imaging software (Essen BioScience, Ann Arbor, MI, USA). Apoptosis was assessed by IncuCyte™ Kinetic Caspase-3/7 Apoptosis Assay Reagent (Essen BioScience, Ann Arbor, MI, USA, 4440).

### Flow cytometry

Apoptosis was assessed by Annexin V/propidium iodide (PI) flow cytometry assay following an incubation of 16 hours with indicated drugs. Cells were then trypsinized and collected, washed with PBS and resuspended in binding buffer. Cells were stained with Annexin-V-FITC and propidium iodide (BD Biosciences, San Jose, CA, USA, 556547) and flow cytometry was undertaken on a BD FACSCanto II flow cytometer (BD Biosciences, San Jose, CA, USA).

The PROTEOSTAT® aggresome detection kit (Enzo Lifesciences, Farmingdale, NY, USA, ENZ-51035-0025) was used to detect aggresome formation following drug treatment at 4 and 18 hours. Following indicated treatment, cells were trypsinized, washed twice with PBS, fixed with 4% formaldehyde and permeabilized with 0.1% Triton X-100. Cells were then stained with the PROTEOSTAT® Aggresome Red Detection Reagent and analyzed by flow cytometry on the FL3 channel of the FACSCanto II.

### Dichlorofluorescein diacetate assay (DCFDA)

ROS (reactive oxygen species) levels were assessed by DCFDA assay (Abcam, Cambridge, MA, USA, ab113851). Cells were seeded in a 96-well plate at 1e4 cells/well. The following day cells were treated with indicated compounds in indicator-free media. At 24 hours post treatment, wells were over-layed with 2x diluted DCFDA reagent, as suggested by the manufacturer’s protocol. Fluorescence was read by microplate reader at Ex/Em = 485/535 nm. A ratio of untreated to treated wells was calculated to determine relative response. Wells were normalized to media only wells to account for background signal.

### Mice

*Pten*^*fl/fl*^*;hSS2* mice (as previously described [22]) received a 10 μL injection of 42 μM TATCre in the hindlimb at 4 weeks of age, to induce expression of SS18-SSX2. A cohort of 7 mice with tumor volumes ranging from 600–1500 mm^3^ were randomly assigned treatment of quisinostat (750 μg/kg) + bortezomib (60 μg/kg) (n = 4) or vehicle control (10% hydroxyl-propyl-β-cyclodextrin/25 mg/mL mannitol/H_2_O) (n = 3). Mice received daily intraperitoneal injections and tumor volumes were measured three times weekly for 21 days. Tumor volumes (mm^3^) were measured using digital calipers and calculated with the formula (length × width^2^)/2 [23]. Tumor volumes at the end of treatment ranged from 900 to 2800 mm^3^. Mice were monitored daily and tumors monitored at least three times weekly during treatment. No toxicity was observed in the treatment regimens of these mice. Mice demonstrating poor mobility, poor feeding/watering behaviors or poor interactions with cage mates, suggestive of ill-health or discomfort, were humanely euthanized. Any tumor burden greater than 10% body mass, that impeded mobility, feeding, or watering or that caused apparent discomfort, or any ulcerated mass or weight loss of more than 20% was also managed by humane euthanasia. Mice were euthanized by exposure to compressed CO^2^ followed by a bilateral thoracotomy. No analgesics or anesthetics were administered during this study. To minimize suffering and distress cages were cleaned regularly, fresh food and water were provided, and mice were housed with siblings for social interaction. Attention was given to routine animal husbandry, and close and frequent observations of mice with tumors or undergoing treatment. This study was carried out in strict accordance with the recommendations in the Guide for the Care and Use of Laboratory Animals of the National Institutes of Health. The protocol was approved by the Institutional Animal Care and Use Committee of the University of Utah (Permit Number: 14–01016).

## Results

### High-throughput drug screen reveals HDAC inhibitors and proteasome inhibitors as potent cytotoxic agents in a panel of synovial sarcoma cell lines

In order to identify drugs effectively targeting synovial sarcoma, a 900 compound high-throughput drug screen representing over 100 drug classes at a concentration of ~1 μM was undertaken in six synovial sarcoma cell lines: SYO-1 (SS18-SSX2), FUJI (SS18-SSX2), Yamato-SS (SS18-SSX1), ASKA-SS (SS18-SSX1), MoJo (SS18-SSX1) and SSR3A1 (murine, SS18-SSX2), as well as negative control cell lines HEK293T (human embryonic kidney) and MCF7 (breast carcinoma). Compounds were scored based on impact on cell viability in and specificity for synovial sarcoma.

**Fig 1:**
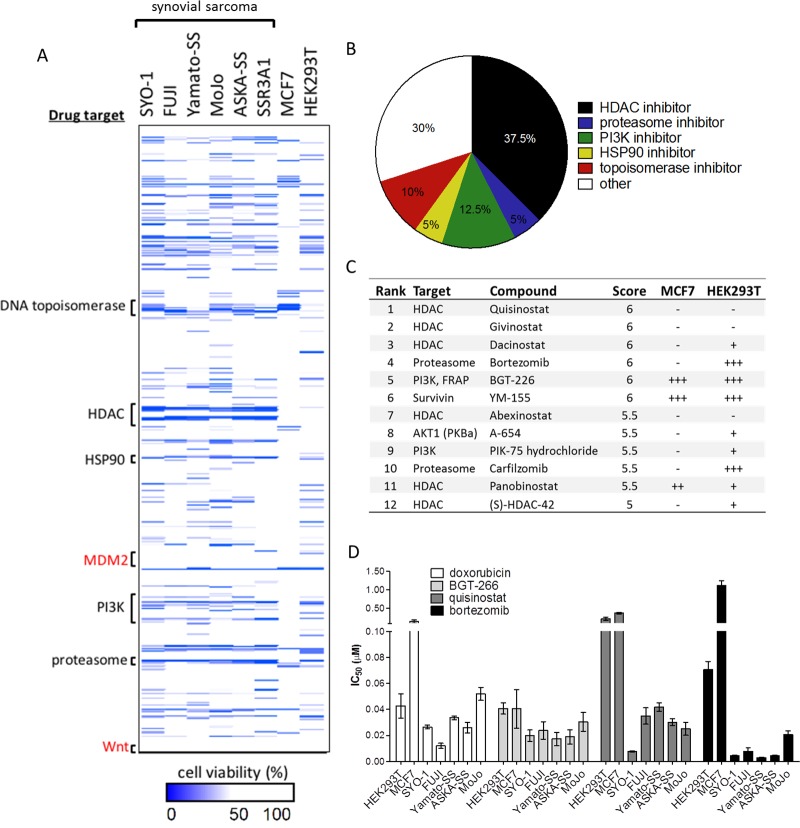
High-throughput drug screen reveals HDAC and proteasome inhibitors as potent drug classes against synovial sarcoma. (A) Compounds resulting in measured relative cell viability of less than 50% are annotated as hits (blue). Y-axis denotes drug target classes arranged in alphabetical order. (B) The top 40 drug screen hits out of the 900 compound screen represented by drug target class, demonstrate HDAC inhibitors as the most abundant hits in the screen. (C) Compounds that brought about greater than 90% decreased relative cell viability were scored as 1 (+++), 75.1–90% as 0.5 (++), 50–75% as 0.25 (+) and less than 50% as 0 (-). Total score across the six cell lines was calculated out of 6. (D) IC_50_ measurements were calculated for drug screen hits quisinostat (HDAC inhibitor), BGT-226 (PI3K/mTOR inhibitor), bortezomib (proteasome inhibitor) as compared with the current standard for synovial sarcoma treatment doxorubicin (cytotoxic DNA/RNA intercalating agent and topoisomerase inhibitor), in a panel of six human SS18-SSX positive cell lines and two control cell lines (HEK293T, MCF7). Error bars signify standard error of mean from conditions performed in triplicate.

There were 84 scored hits out of the 900 library compounds. Of the top 40 scored compounds, 15 (37.5%) of the most potent drugs were HDAC inhibitors (Fig 1A and 1B). Both of the proteasome inhibitors in the drug library scored in the top ten (Fig 1C). Also of note, PI3K inhibitors comprised five (12.5%) of the top hits. The most potent drugs were further validated in dose-response studies, and IC_50_ values in all cell lines were calculated (Fig 1D). The novel second generation pan-HDAC inhibitor quisinostat (JNJ-26481585) is the most potent and specific of the studied HDAC inhibiting compounds in synovial sarcoma cell lines. Proteasome inhibitors bortezomib and carfilzomib are similarly potent in decreasing cell viability in synovial sarcoma. Drug classes that have little effect on synovial sarcoma at the studied doses included modulators of the Wnt pathway and MDM2 inhibitors (Fig 1A and S1 Fig).

### HDAC inhibition by quisinostat derepresses tumor suppressor gene targets of SS18-SSX in synovial sarcoma

**Fig 2:**
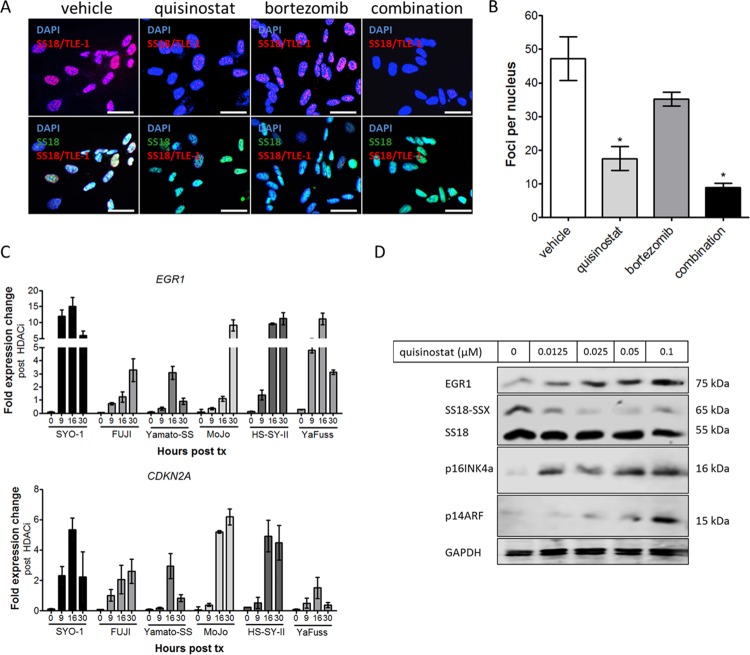
Quisinostat-mediated HDAC inhibition results in a dissociation of the driving complex in synovial sarcoma. (A, B) Proximity ligation assay of SS18-SSX/TLE1 nuclear signal demonstrates a significant decrease in detectable protein co-localization following HDAC inhibition in SYO-1 synovial sarcoma cells. (C) Quisinostat treatment at 0.025 μM reactivates targets of SS18-SSX-mediated gene repression, *EGR1* and *CDKN2A*, in six human synovial sarcoma cell lines. (D) Expression of EGR1, p16INKa and p14ARF (*CDKN2A*) protein levels increase with increasing concentrations of quisinostat, concomitant with a decrease in SS18-SSX protein levels. GAPDH was used as a loading control. Scale bars in panel A represent 20 μm. Statistical significance compared to vehicle treatment controls was determined by Student t test: * denotes *p* < 0.05. Error bars represent standard error of mean from conditions performed in triplicate.

To demonstrate the effects of HDAC inhibition on gene expression in synovial sarcoma, RNA and protein analyses were undertaken following treatment with quisinostat in six SS18-SSX-containing cell lines. In accordance with previous reports [11], the key association of SS18-SSX with TLE1 is lost following treatment with quisinostat, with or without the addition of proteasome inhibitor bortezomib (Fig 2A and 2B). To assess whether there are any superadditive effects in this model, an interaction term between quisinostat and bortezomib was added in a linear regression model (foci per nuclei as the dependent variable, and quisinostat/bortezomib treatment as independent variables). The disruption of the SS18-SSX/TLE1 proximity interaction is additive but not significantly synergistic in the combination treatment arm, as demonstrated by the synergy interaction statistical analysis (p = 0.66). RNA expression at gene targets previously shown to be directly repressed by SS18-SSX activity [9] is reactivated by quisinostat treatment in six synovial sarcoma cell lines (Fig 2C). Following quisinostat treatment, tumor suppressors EGR1 and p16INK4a/ p14ARF (*CDKN2A)* are reactivated at the protein level, whereas SS18-SSX oncoprotein levels were reduced (Fig 2D).

### HDAC inhibition synergizes with proteasome inhibition to decrease synovial sarcoma cell viability

**Fig 3:**
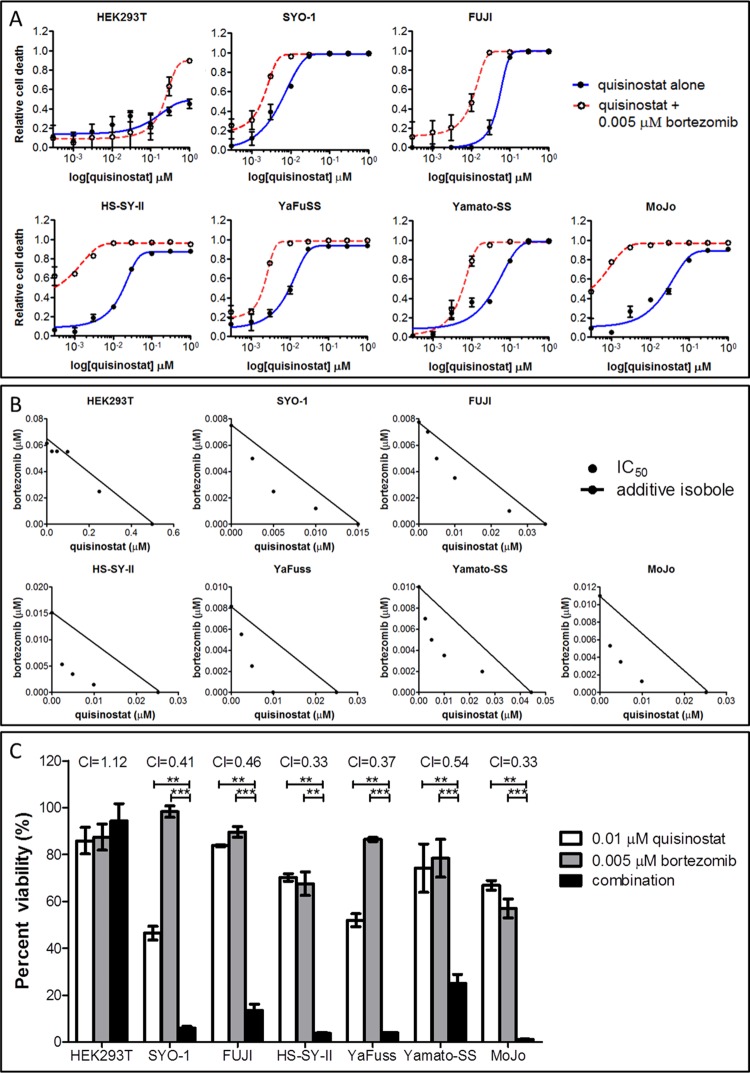
HDAC inhibition by quisinostat synergizes with proteasome inhibition to decrease synovial sarcoma cell viability. (A) In all synovial sarcoma cell lines, but not HEK293T controls, the addition of 0.005 μM of bortezomib results in a downshift of approximately a full log of quisinostat, decreasing the amount of drug required to achieve the same effect as the HDAC inhibitor alone. (B) Isobologram analysis demonstrates synergy of these drug classes in synovial sarcoma cell lines (but not HEK293T controls), as increasing concentration combinations fall below the additive isoboles. (C) Combination index (CI) values calculated for the combination of bortezomib and quisinostat in synovial sarcoma are significantly less than 1, indicating synergy of the compounds is occurring in all six synovial sarcoma cell lines (but not HEK293T controls). Isobolograms and CI values were calculated using the Chou-Talalay-designed program CompuSyn. Statistical significance compared to vehicle treatment controls was determined by Student t test: * denotes *p* < 0.05; ** denotes *p* < 0.01; *** denotes *p* < 0.001. Error bars represent standard error of mean from conditions performed in triplicate.

To investigate the potential of drug combinations building on an HDAC inhibitor backbone, we tested quisinostat in combination with proteasome inhibitors, the next most potent drug class revealed from the drug screen. Synovial sarcoma cells were assayed for relative cell viability, in comparison with the vehicle control (0.1% DMSO) by way of a dose curve of quisinostat, with and without the addition of 0.005 μM of bortezomib. With bortezomib, a significant increase in normalized response is observed: in the presence of low-dose proteasome inhibition, quisinostat dosage can be reduced by an order of magnitude yet achieve the same effect (Fig 3A). This combinatorial benefit is not observed in control cell line HEK293T at the studied doses. Similar studies were also undertaken with HDAC and PI3K inhibitor combinations, but with this combination no synergy was observed in the synovial sarcoma cell lines at the studied doses.

**Fig 4:**
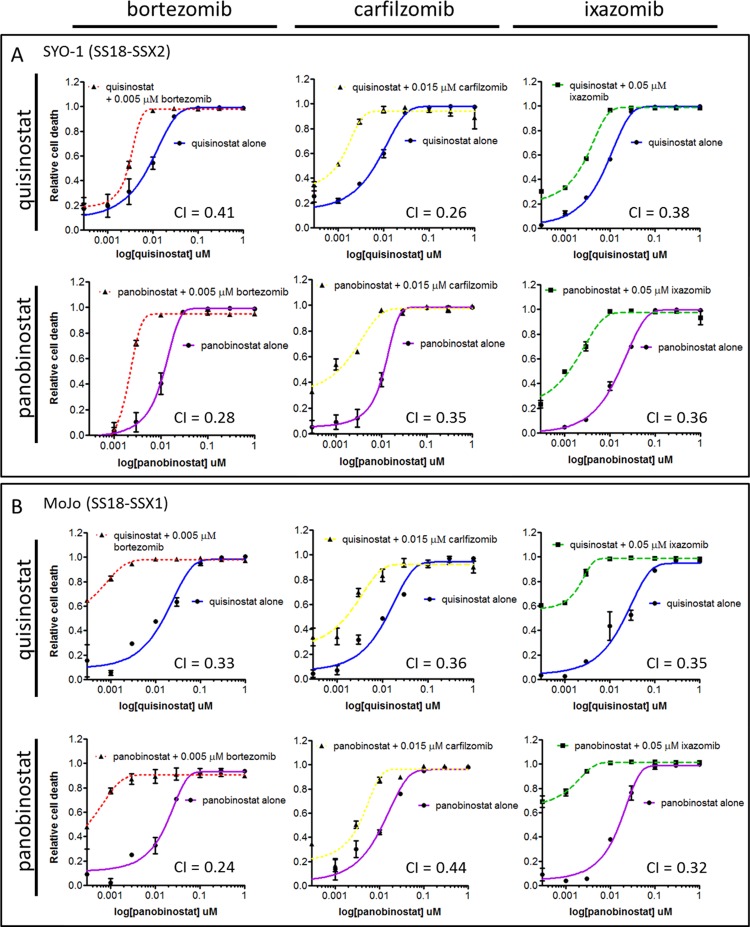
The synergistic effect of HDAC and proteasome inhibition is consistent within each drug class. Additional compounds of each drug class were tested in combinational synergy studies. Quisinostat, panobinostat (pan-HDAC inhibitors), and bortezomib, carfilzomib and ixazomib (proteasome inhibitors) were studied in all combinations in the SYO-1 (A) and MoJo (B) SS18-SSX containing cell lines. CI values are less than 1 in these synovial sarcoma cell lines. CI values were calculated using the Chou-Talalay-designed program CompuSyn. Error bars represent standard error of mean from conditions performed in triplicate.

Synergy was assessed by isobologram analysis (Fig 3B), in which the combination isoboles at a constant ratio and at six increasing concentrations fall below the additive isobole, indicating synergism of the effects of the two compounds. Combination index (CI) values were calculated using the Chou-Talalay methodology and found to be less than 1, demonstrating synergy of the combination in all six tested synovial sarcoma cell lines (Fig 3C). The combination of bortezomib and quisinostat brings about significantly stronger anti-proliferative effects than either drug alone, at low nanomolar doses, across the panel of SS18-SSX-containing cell lines. This synergistic effect is consistently observed with additional compounds of each drug class, including the pan-HDAC inhibitor panobinostat, and proteasome inhibitors carfilzomib and ixazomib (Fig 4A and 4B). CI values with combinations of these agents are consistently found to be less than 1 in synovial sarcoma cell lines.

### HDAC inhibition disrupts aggresome formation in response to proteasome inhibition

**Fig 5:**
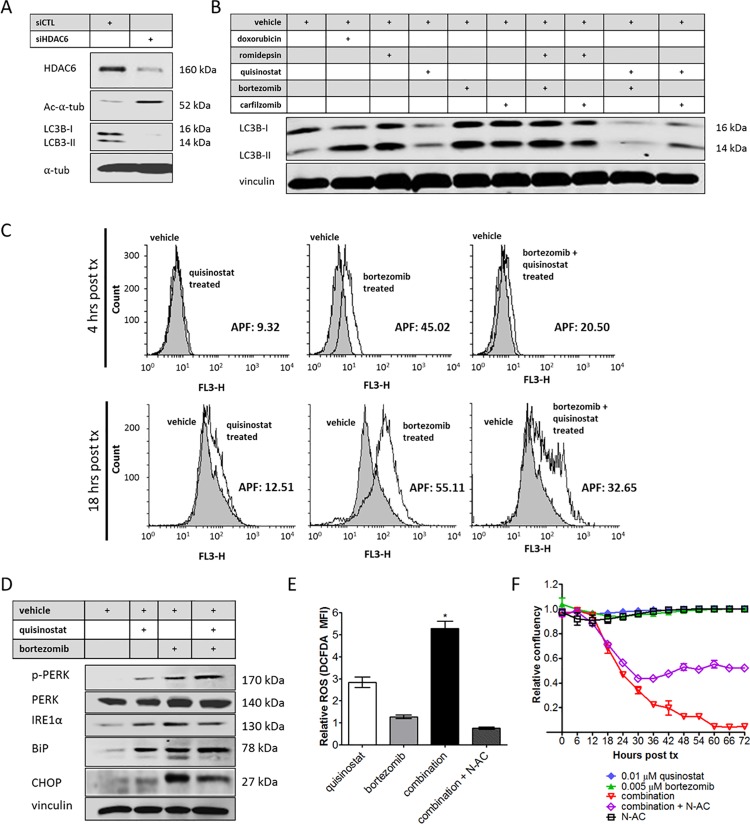
HDAC inhibition prevents aggresome formation in response to proteasome inhibitors, and combination treatment leads to endoplasmic reticulum stress. (A) Knockdown of HDAC6 results in decreased levels of LC3B in the SYO-1 synovial sarcoma cell line. (B) Proteasome inhibition as well as treatment with class I HDAC inhibitor romidepsin increases in LC3B levels, whereas quisinostat treatment decreases protein levels following treatment with proteasome inhibitors bortezomib or carfilzomib. (C) Aggresome formation is induced by bortezomib at 4 hours post treatment by PROTEOSTAT® staining analyzed by flow cytometry, an effect that is abrogated by quisinostat. The aggresome propensity factor (APF) is significantly decreased with the addition of quisinostat in the context of proteasome inhibition. (D) Endoplasmic reticulum stress markers are expressed following combination treatment and (E) a significant increase in ROS activity is measured by mean fluorescence intensity (MFI) of DCFDA, as compared with vehicle treated cells. ROS activation is abrogated by N-acetylcysteine (N-AC) treatment at 10 mM. (F) Cell death is rescued with N-AC by ~50%. Statistical significance compared to vehicle treatment controls was determined by Student t test: * denotes *p* < 0.05. Error bars represent standard error of mean from conditions performed in triplicate. Vinculin or α-tubulin was used as a loading control for protein analysis.

To investigate potential mechanisms of synergy, the effect of HDAC inhibition on aggresome formation was assessed. Proteasome inhibition is known to bring about aggresome formation as a response to accumulating misfolded proteins, a mechanism facilitated by HDAC6 activity [24, 25]. Silencing of HDAC6 by siRNA brings about a decrease in LC3B protein levels, a marker of aggresome formation (Fig 5A). When treated with quisinostat, LC3B protein levels are similarly decreased, whereas the pure class I HDAC inhibitor romidepsin (which does not inhibit HDAC6) enhances LC3B expression (Fig 5B). When treated with proteasome inhibitors bortezomib, carfilzomib, or a combination of proteasome inhibition and romidepsin, LC3B levels are increased. When used in combination with proteasome inhibition, treatment with quisinostat remains able to elicit a decrease in LC3B levels, indicating inhibition of aggresome formation attributable to its impact on HDAC 6 activity (Fig 5B).

By flow cytometry, aggresome formation is demonstrated to be induced by proteasome inhibition (Fig 5C). HDAC inhibition by quisinostat is consistently found to inhibit the aggresome response. Aggresome propensity factors (APF) were calculated using the Enzo Life Sciences Aggresome detection assay, wherein an APF of greater than 25 is indicative of aggresome formation in response to proteasome inhibition. With the addition of quisinostat, the APF is significantly decreased when compared with bortezomib alone in SYO-1 synovial sarcoma cells at 4 and 18 hours post treatment (Fig 5C).

### HDAC and proteasome inhibitor combination treatment leads to ER stress and increased ROS levels

In order to further examine the role of the HDAC/proteasome inhibitor drug combination in augmenting stress in synovial sarcoma, the unfolded protein response was investigated. Bortezomib has been shown previously to bring about endoplasmic reticulum (ER) stress in cells due to misfolded protein accumulation leading to high levels of reactive oxygen species (ROS) [26, 27]. The combination of quisinostat and bortezomib elicits high expression of ER stress markers in synovial sarcoma cell lines, as demonstrated by the activation of: phosphorylated-PERK (slows translation [28]), IRE1α (unfolded protein response element involved in ER chaperone upregulation and stress recovery [29]), BiP (molecular chaperone [30]), phosphorylated-JNK (c-Jun N-terminal kinases, activated in response to cellular stress [31]) and CHOP (DDIT3, pro-apoptotic protein [32]) (Fig 5D). ROS activity is also significantly increased in response to the combination of HDAC and proteasome inhibition (Fig 5E). ROS levels can be experimentally abrogated by the addition of antioxidant N-Acetylcysteine (N-AC) during drug treatment. Addition of N-AC rescued SYO-1 cells from HDAC inhibitor/proteasome inhibitor-mediated cell death by approximately 50% (Fig 5F).

### The combination of HDAC and proteasome inhibition leads to apoptosis in synovial sarcoma cells and inhibition of tumor growth in a synovial sarcoma conditional mouse model

**Fig 6:**
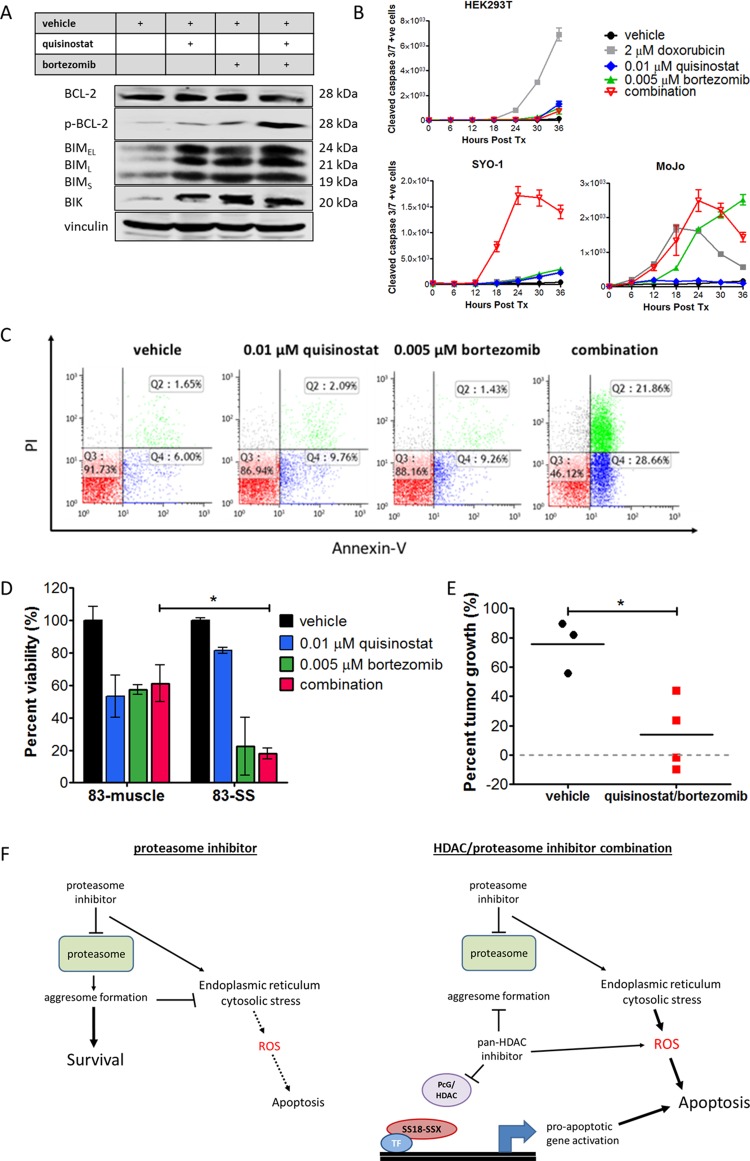
HDAC and proteasome inhibition leads to apoptosis via pro-apoptosis protein activation, ROS production and caspase activation. (A) Pro-apoptotic proteins BIM and BIK are upregulated by both quisinostat and bortezomib, and the drug combination elicits phosphorylation of anti-apoptotic protein BCL-2 in SYO-1 cells. (B) Cleavage of caspase 3 occurs following treatment with the drug combination in synovial sarcoma cell lines, demonstrated by staining with IncuCyte™ Kinetic Caspase-3/7 Apoptosis Assay Reagent, (C) inducing significant apoptosis as confirmed by Annexin-V/PI staining in the SYO-1 cell line (Q3: live, Q2: necrotic/late apoptotic, Q4: early apoptotic). (D) The low-dose quisinostat/bortezomib drug combination brings about a significant decrease in the viability of primary synovial sarcoma cells (83-SS) as compared to matched normal muscle cells derived from the same patient (83-muscle). Two-way ANOVA indicated a significant interaction between cell type and response to the drug combination (*p* < 0.05). (E) Tumor growth in a murine model of synovial sarcoma was significantly reduced by day 21 with the quisinostat/bortezomib combination treatment, as compared to the vehicle only control. (F) Taken together, the combination of HDAC and proteasome inhibitors results in dissociation of the SS18-SSX driving complex as well as aggresome inhibition, ER stress and ROS production, leading to apoptosis induction in synovial sarcoma. Statistical significance compared to vehicle treatment controls was determined by Student t test or two-way ANOVA where indicated: * denotes *p* < 0.05. Error bars represent standard error of mean from conditions performed in triplicate. Vinculin was used as a loading control for protein analysis.

Pro-apoptotic proteins BIM and BIK are upregulated by both quisinostat and bortezomib treatments, and combination treatment elicits phosphorylation of anti-apoptotic protein BCL-2 (Fig 6A). Cleaved caspase 3/7 induction is significantly increased over time in synovial sarcoma cell lines SYO-1 and MoJo following low dose treatment of the quisinostat/bortezomib combination, while only doxorubicin had a significant effect on the HEK293T cells, as measured by IncuCyte™ Kinetic Caspase-3/7 Apoptosis Assay Reagent (Fig 6B). A significant increase in Annexin-V staining is observed when the drugs are used in combination, to an extent greater than that of either drug alone (Fig 6C). Cell viability was also significantly decreased by the low-dose quisinostat/bortezomib combination in primary patient synovial sarcoma cells (83-SS) as compared with their matched normal muscle cells (83-muscle) (Fig 6D).

To demonstrate the efficacy of the drug combination *in vivo*, quisinostat and bortezomib were tested for their effect on tumors in a murine model of synovial sarcoma harboring the SS18-SSX2 translocation. Doses used were similar or less than that of previous animal studies of this drug combination in multiple myeloma, in which single drug arms showed little to no efficacy, while the combination decreased tumor burden [33]. Due to the large number of drug dose combinations and replicates required, synergy is more practically assessed in cell line experiments. The mouse model of synovial sarcoma is used in this study to confirm the activity of the best drug combination. Tumor growth is significantly reduced by treatment with the combination of quisinostat and bortezomib, as compared with vehicle treated mice over a period of 21 days (Fig 6E and S2 Fig).

## Discussion

Current cytotoxic therapies offer limited benefit in synovial sarcoma; improved, more targeted therapeutics are needed. As the sole driving cytogenic event, t(X;18) generates the SS18-SSX protein complex that leads to the distinct phenotype of this cancer and provides an attractive target for drug intervention. In this study we show that HDAC inhibition by quisinostat is able to dissociate the driving complex in synovial sarcoma, resulting in reactivated expression of tumor suppressors otherwise repressed by SS18-SSX. Furthermore, we demonstrate that apoptosis is enhanced following HDAC inhibition when used in combination with a proteasome inhibitor. This combination triggers ER stress, phosphorylation of the overexpressed anti-apoptotic protein BCL-2, and activation of pro-apoptotic proteins BIM and BIK. Together these drug classes synergize at low nanomolar doses to induce synovial sarcoma cell death.

We have previously shown that SS18-SSX interacts with its partners at promoters recognized by ATF2, repressing transcription at critical tumor suppressor loci including *EGR1* and *CDKN2A* [9]. HDAC inhibitors dissociate the driving complex and reactivate *EGR1* and p16INK4a/p14ARF *(CDKN2A)* expression in synovial sarcoma; we confirm this occurs with treatment by quisinostat, a newer HDAC inhibitor that was the top hit in our compound screen. EGR1 is an important regulator of several tumor suppressors including PTEN [34] and p53 [35], and reports have shown EGR1 directly transactivates expression of BIM [36] and mediates c-MYC-induced apoptosis in cooperation with p14ARF [37]. In addition, regulation by p16INK4a and p14ARF is important for cell cycle arrest via inhibition of CDK4/6 [38], as well as for apoptosis induction via upregulation of BIK [39] and p53-dependent signaling [40]. HDAC inhibition may therefore impede SS18-SSX-mediated deregulation at these loci, allowing for reactivation of normal cell cycle regulation and apoptotic pathways.

Proteasome inhibition has been shown to induce ER stress due to misfolded protein accumulation; aggresome formation functions as a cytoprotective mechanism in this system by packaging misfolded protein aggregates for lysosomal degradation [24, 41]. With the addition of HDAC6 inhibition by quisinostat, shuttling of misfolded proteins to aggresomes is blocked, circumventing this resistance mechanism [25]. Excessive ER stress resulting from misfolded protein accumulation leads to elevated ROS levels and initiation of apoptosis [42]. Phosphorylation of BCL-2, deactivating this important anti-apoptotic regulator [43, 44], is also found to occur following the quisinostat/proteasome inhibitor combination treatment, resulting in a pro-apoptotic shift not seen with doxorubicin treatment in synovial sarcoma [45].

Proteasome inhibition has also been established as a specific vulnerability of Ewing sarcoma (*EWS-FLI1*) [46], suggesting a particular susceptibility of this related translocation-associated sarcoma to proteasome inhibitor drug intervention. Additional reports have characterized BIM and BIK activation as key components of proteasome inhibitor induced apoptosis, as they are found to be significantly upregulated and stabilized following bortezomib treatment [47, 48].

Recent studies have demonstrated a compensatory role for proteasome activity in response to HDAC inhibition. A genome-wide screen revealed RAD23B, a protein that shuttles ubiquitinated proteins to the proteasome, is a biomarker for sensitivity to HDAC inhibition in cutaneous T-cell lymphoma [49, 50], and this relationship has been further demonstrated to also apply in sarcomas [51]. These studies suggest RAD23B is regulated by HDAC activity, and upon HDAC inhibition the shuttling function is over-activated and may sensitize cells to proteasome inhibition, providing further support for a combinatorial treatment strategy combining these drug classes.

The combination of quisinostat and bortezomib has been evaluated in a phase-Ib clinical trial in multiple myeloma, from which an acceptable safety profile was observed, and partial response or better was achieved in 15 out of 17 patients [52]. The combination of the pan-HDAC inhibitor vorinostat with bortezomib has also been shown to be active in refractory multiple myeloma, offering a third-line treatment modality in difficult and relapsed cases [53]. Our study shows that in synovial sarcoma models, the combination of HDAC and proteasome inhibition functionally dissociates the oncogenic driving complex, reactivates tumor suppressor gene expression, and elicits ER stress, elevated ROS levels, and pro-apoptotic factor induction, resulting in significant cell death even at very low drug concentrations (Fig 6F). The present study demonstrates the potential value of the combining HDAC and proteasome inhibitors as a treatment for synovial sarcoma.

## Supporting Information

S1 Fig Complete list of drug screen results in six synovial sarcoma cell lines. Synovial sarcoma: SYO-1, FUJI, Yamato-SS, ASKA-SS, MoJo, SSR3A1; control cell lines: MCF7 (human cervical carcinoma), HEK293T (human embryonic kidney). Drug compound libraries comprised of tool compounds (Ontario Institute of Cancer Research) and epigenetic modifiers (Cayman Biochemical, Item 11076) were screened in a high-throughput (96-well pin tool) format. Compounds resulting in measured relative cell viability of less than 50% are annotated as hits (blue). Y-axis denotes compound names and drug target classes arranged in alphabetical order. Viability heatmap was created using the Gene-E software program.
(PDF)

S2 Fig Tumor growth is significantly reduced by treatment with the combination of quisinostat and bortezomib, as compared with vehicle treated mice over a period of 21 days. Tumor-bearing mice were randomly assigned to groups treated with quisinostat (750 μg/kg) + bortezomib (60 μg/kg) (n = 4) or with vehicle control (10% hydroxyl-propyl-β-cyclodextrin/25 mg/mL mannitol/H_2_O) (n = 3). Mice received daily intraperitoneal injections and tumor volumes were measured three times weekly for 21 days. Tumor growth was significantly greater in the untreated group than in the treated group, consistently over the time period. Statistical significance was determined by one-way ANOVA test: *** denotes *p* < 0.001. Error bars represent standard error of mean.
(TIF)
