# Supplementary figures and images for: HDAC and Proteasome Inhibitors Synergize to Activate Pro-Apoptotic Factors in Synovial Sarcoma
Source: PLoS One. 2017 Jan 5;12(1):e0169407. doi: 10.1371/journal.pone.0169407 (PMC5215898; doi:10.1371/journal.pone.0169407)

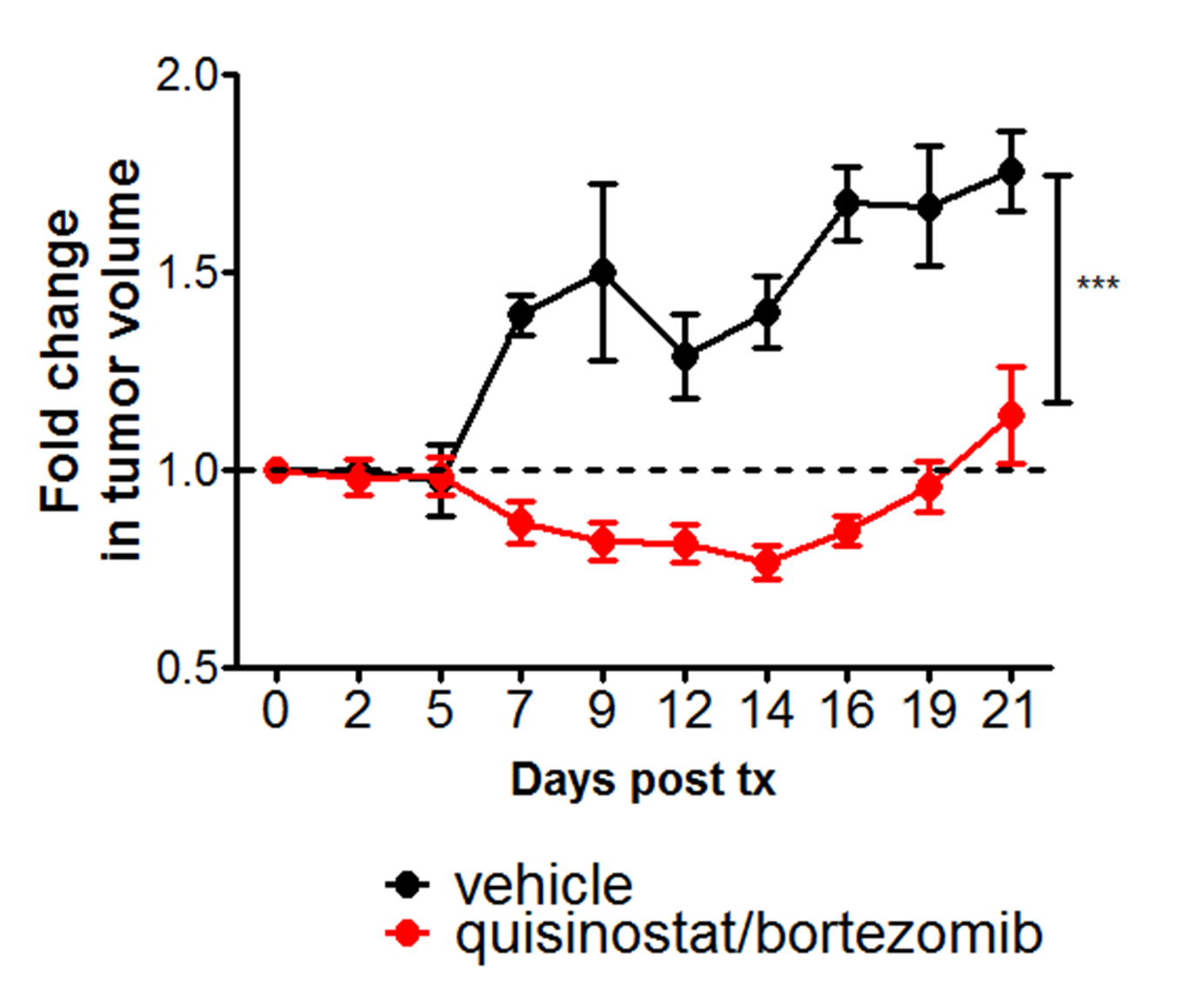

Supplement: S2 Fig — Tumor-bearing mice were randomly assigned to groups treated with quisinostat (750 μg/kg) + bortezomib (60 μg/kg) (n = 4) or with vehicle control (10% hydroxyl-propyl-β-cyclodextrin/25 mg/mL mannitol/H2O) (n = 3). Mice received daily intraperitoneal injections and tumor volumes were measured three times weekly for 21 days. Tumor growth was significantly greater in the untreated group than in the treated group, consistently over the time period. Statistical significance was determined by one-way ANOVA test: *** denotes p < 0.001. Error bars represent standard error of mean. (TIF) [file pone.0169407.s002.tif]
