# Supplementary material for: HDAC and Proteasome Inhibitors Synergize to Activate Pro-Apoptotic Factors in Synovial Sarcoma
Source: PLoS One. 2017 Jan 5;12(1):e0169407. doi: 10.1371/journal.pone.0169407 (PMC5215898; doi:10.1371/journal.pone.0169407)
Supplement: S1 Fig — Synovial sarcoma: SYO-1, FUJI, Yamato-SS, ASKA-SS, MoJo, SSR3A1; control cell lines: MCF7 (human cervical carcinoma), HEK293T (human embryonic kidney). Drug compound libraries comprised of tool compounds (Ontario Institute of Cancer Research) and epigenetic modifiers (Cayman Biochemical, Item 11076) were screened in a high-throughput (96-well pin tool) format. Compounds resulting in measured relative cell viability of less than 50% are annotated as hits (blue). Y-axis denotes compound names and drug target classes arranged in alphabetical order. Viability heatmap was created using the Gene-E software program. (PDF) [file pone.0169407.s001.pdf]

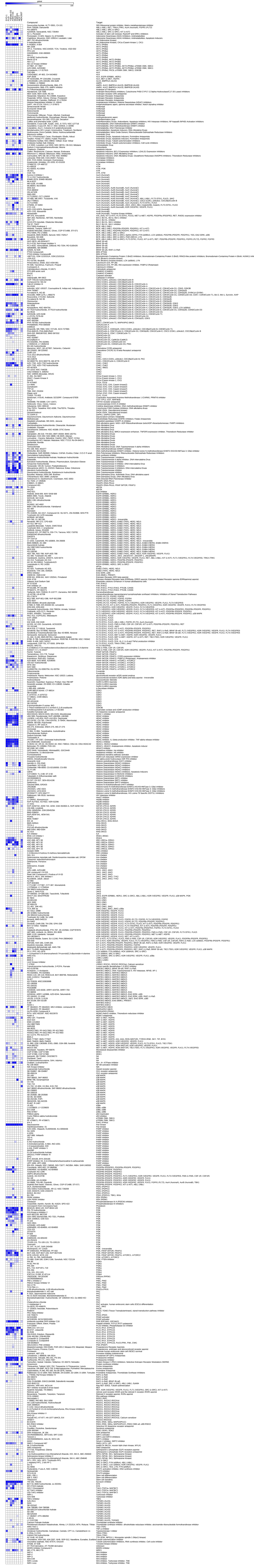
global
0
50
100
SYO-1
FUJI
Yamato-SS
ASKA-SS
MoJo
SSR3A1
MCF7
HEK293T
Compound
Target
